# Supplementary material for: Tannin-Derived Hard Carbon for Stable Lithium-Ion Anode
Source: Molecules. 2022 Oct 18;27(20):6994. doi: 10.3390/molecules27206994 (PMC9611679; doi:10.3390/molecules27206994)
Supplement: Supplementary file 1 [file molecules-27-06994-s001.zip › molecules-1966292-supplementary.pdf]

Supporting Information

Tannin-derived hard carbon for stable lithium-ion anode

He Ming-Jun[1,2,#], Xu Lai-Qiang[1,#], Feng Bing[1], Hu Jin-Bo[1,3,*], Chang Shan-Shan[1], Liu Gong-Gang[1,3,*], Liu Yuan[1], and Xu Bing-Hui[4]

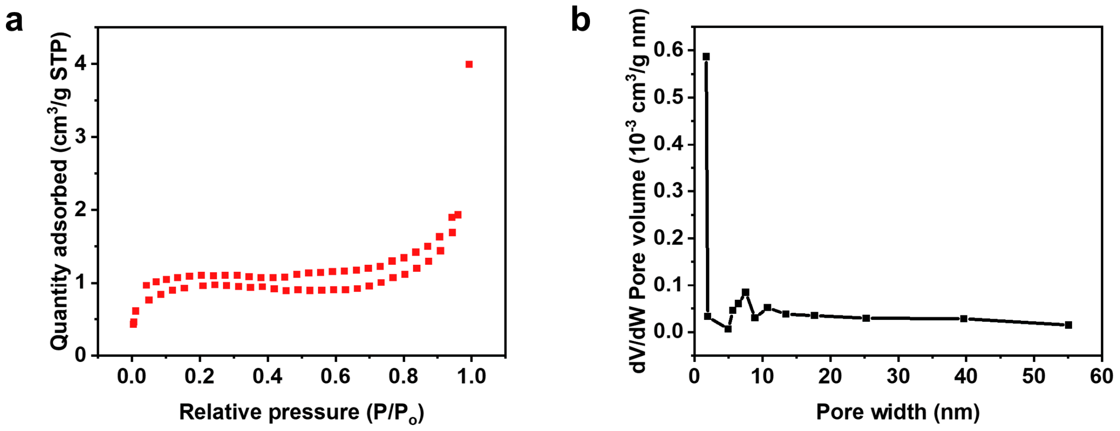

Figure S1. (a) Nitrogen adsorption and desorption curve of HC. **(b)** Pore size distribution curve of HC.

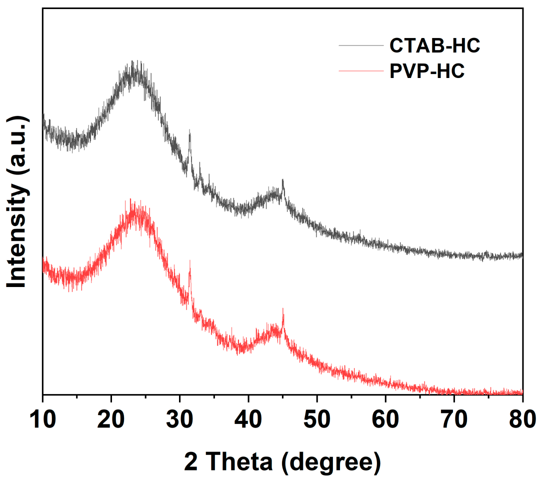

Figure S2. XRD patterns of CTAB-HC and PVP-HC.

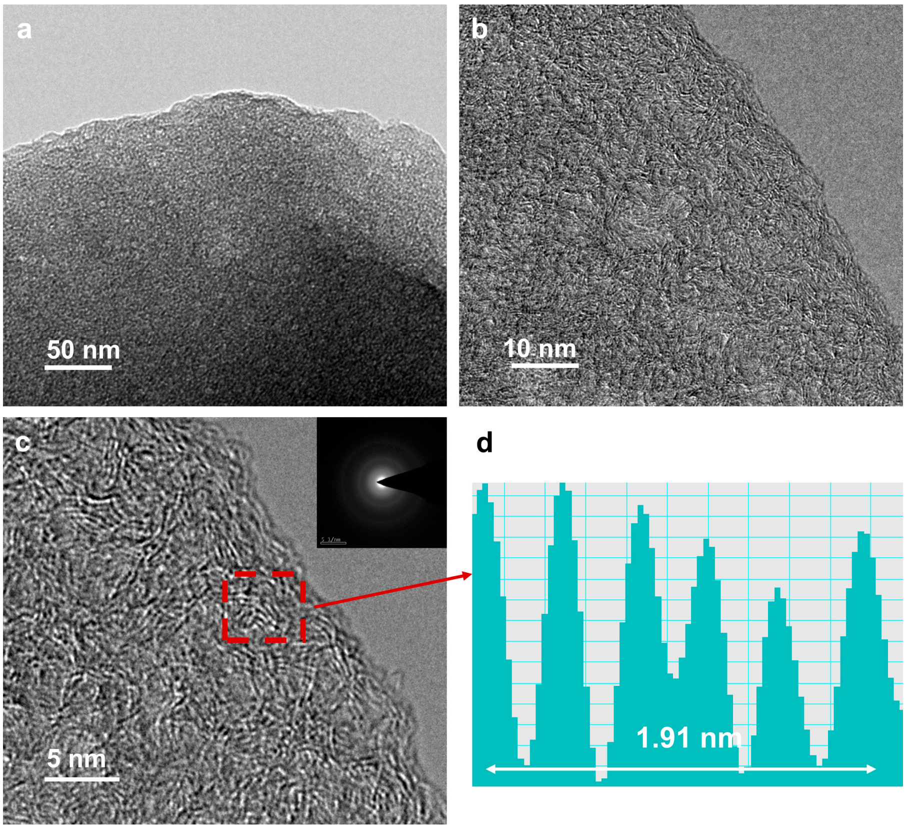

Figure S3. (a-b) TEM images of the hard carbon. **(c)** HRTEM image of the hard carbon (inset image: SAED image of HC). **(d)** Layer spacing of the hard carbon obtained by inverse fourier transform.

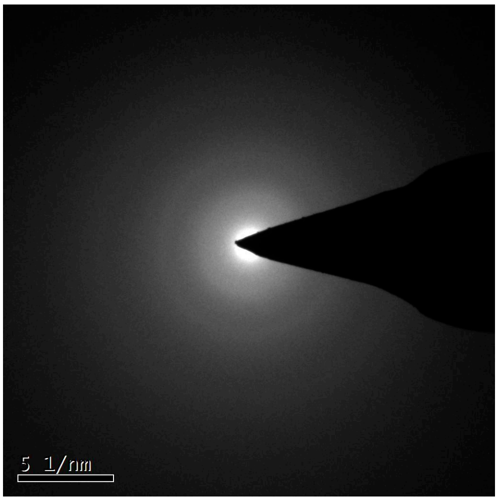

Figure S4. SAED image of PVP-HC.

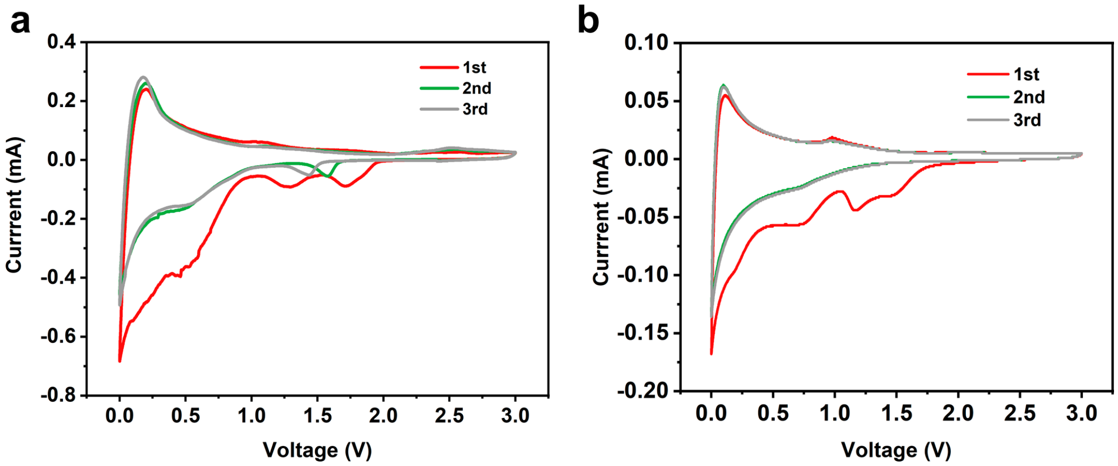

Figure S5. Cyclic voltammetric curves **(a)** HC, **(b)** CTAB-HC.

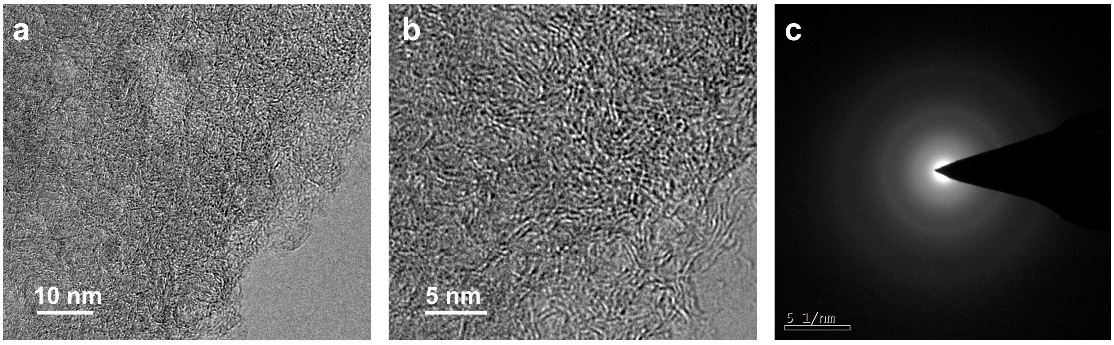

Figure S6. (a) TEM image, **(b)** HRTEM , **(c)** SAED image of HC after cycling.
